# Radionuclide Methods in the Diagnosis of Sacroiliitis in Patients with Spondyloarthritis: An Update

**DOI:** 10.5041/RMMJ.10264

**Published:** 2016-10-31

**Authors:** Karina Zilber, Miguel Gorenberg, Doron Rimar, Nina Boulman, Lisa Kaly, Michael Rozenbaum, Itzhak Rosner, Gleb Slobodin

**Affiliations:** 1 Rheumatology Unit, Bnai Zion Medical Center, Haifa, Israel; 2 Department of Nuclear Medicine, Bnai Zion Medical Center, Haifa, Israel; 3 Ruth and Bruce Rappaport Faculty of Medicine, Technion, Haifa, Israel

**Keywords:** Nuclear medicine, sacroiliitis, scintigraphy, spondyloarthritis, technetium, tomography

## Abstract

Sacroiliitis, inflammation of the sacroiliac joint (SIJ), is the hallmark of ankylosing spondylitis and spondyloarthritis (SpA) in general. The arsenal of recommended diagnostic modalities for imaging of the SIJ is scanty and, in practice, includes only conventional X-rays and magnetic resonance imaging (MRI). This review suggests that bone scintigraphy, particularly single-photon emission computed tomography (SPECT) with calculation of indices, or SPECT in combination with low-dose computed tomography (CT) can be a sensitive and specific tool for the diagnosis of sacroiliitis and can be used as part of the individualized approach to the diagnosis of axial SpA. In addition, [^18^F]fluoride positron emission tomography (PET)/CT imaging and immunoscintigraphy, using labeled monoclonal anti-cytokine antibodies, are promising methods of current scientific interest in this field.

## INTRODUCTION

Sacroiliitis, inflammation of the sacroiliac joint (SIJ), the hallmark of ankylosing spondylitis (AS), may be present in the course of a variety of other rheumatic and non-rheumatic disorders as well.^1^ Clinical features of sacroiliitis include low back and buttock pain, tenderness of the involved SIJ on palpation, and positive sacroiliac pain provocation tests, such as FABER (Flexion, ABduction, and External Rotation) or Gaenslen’s maneuvers. Intense and disabling sacroiliac pain can be seen in patients with acute sacroiliitis, while insidious pain with night worsening, morning stiffness and improvement after exercise is more typical in patients with AS and other chronic rheumatic conditions.^2^ However, these clinical features are not pathognomonic for sacroiliitis, and, in practice, only a minority of patients presenting with the above-mentioned complaints suffer from sacroiliitis. Hence, imaging has assumed a crucial role in the diagnosis of sacroiliac involvement in the disease process.

Presently, available methods for the imaging of sacroiliac joints are limited. Conventional radiography lacks sensitivity, particularly at the early stages of sacroiliitis; computed tomography (CT) scanning is not well validated for the diagnosis of sacroiliitis, demonstrates only structural changes, i.e. erosions and ankylosis, and is associated with relatively high radiation doses; and while magnetic resonance imaging (MRI) is able to show active inflammation before the consequent structural lesions develop, its sensitivity for detection of such changes is only moderate, at best only up to 70%.^3^,^4^

Bone scintigraphy has been used to detect sacroiliitis for years. It has been appreciated for decades as a valuable method for the ascertainment of acute sacroiliitis, but its accuracy in the diagnosis of chronic inflammation in SIJ has always been questionable. A review on the performance of scintigraphy in assessing sacroiliitis in patients with AS, published in 2008, suggested that scintigraphy of the sacroiliac joints is at most of limited diagnostic value for this diagnosis.^5^ That review had a great impact and was the final step in establishing the singular dominance of MRI in the imaging of sacroiliitis. Only a few studies have aimed to investigate further the potential of radionuclide methods for the diagnosis of SIJ involvement since then. We believe, however, that at least some of these studies are important for the present and future practice of medicine. Accordingly, the aim of the present paper is to review and summarize the published medical literature since 2008 on the usage of radionuclide techniques for the diagnosis of sacroiliitis and spondyloarthritis (SpA) in general.

## METHODS

A systematic PubMed search using the keywords “sacroiliitis” or “spondyloarthritis” or “ankylosing spondylitis” in combination with “bone scintigraphy” or “bone scan” was performed and relevant articles extracted and critically assessed. The data were further summarized and presented.

## RESULTS

### Bone Scintigraphy with Technetium-99m-labeled Methylene Diphosphate

This method can demonstrate increased radionuclide uptake in the areas of accelerated bone turnover due to any cause, including inflammation. During the examination of the SIJ, the intensity of radionuclide uptake in the area of interest is compared to an adjacent background structure, usually sacrum, allowing quantitative interpretation of the result. Due to the low sensitivity of about 50% and calculated positive likelihood ratio of about 3 for the diagnosis of sacroiliitis, this method of SIJ imaging was suggested to be of limited, if any, value by Song et al., in 2008.^5^ Of interest, however, even then, in patients with earlier stages of radiographic sacroiliitis, those where the real need in the confirmatory imaging exists, the sensitivity of bone scan was calculated as closer to 60%. In addition, as a possible limitation of the analysis, MRI was chosen as the gold standard for the assessment of bone scan performance for SIJ imaging in some of the reviewed studies, while it is well accepted today that MRI itself has sensitivity of only about 70% for the diagnosis of sacroiliitis.^3^–^5^ Subsequently, Song et al. reported in 2010 their own retrospective study on the performance of radionuclide scan in 207 patients with chronic low back pain for the diagnosis of SpA.^6^ In this study, where the rheumatologist’s diagnosis was chosen as the gold standard, sensitivities of scintigraphy for any (unilateral or bilateral), bilateral, and isolated unilateral sacroiliitis were 64.9%, 40.2%, and 24.7%, respectively. Respective specificities were 50.5%, 57.7%, and 92.8%, resulting in likelihood ratios of 1.3, 1.0, and 3.4.^6^ In another study, specificity of quantitative scintigraphy in 30 patients with MRI-positive non-radiographic SpA was calculated as 100%, while sensitivity of the method was only 32%.^7^ Finally, the diagnostic potential of bone scintigraphy to elucidate unappreciated articular and entheseal involvement in addition to the imaging of the SIJ in SpA was examined and found useful in the study of Gheita et al.,^8^ thus offering an additional benefit of this modality.

### Single-Photon Emission Computed Tomography

**Figure 1:**
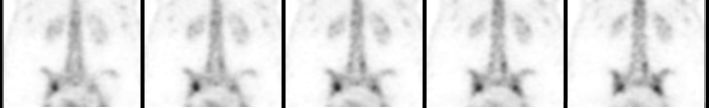
Technetium-99m-MDP SPECT in Ankylosing Spondylitis. Technetium-99m-MDP SPECT shows increased uptake in both sacroiliac regions in a patient with ankylosing spondylitis.

It has been known for years that single-photon emission computed tomography (SPECT) imaging increases sensitivity of bone scintigraphy, allowing slice-by-slice three-dimensional radionuclide uptake analysis. This possibility can be particularly useful in the study of the SIJ, where complex anatomy is probably the main cause of low accuracy of both plain radiography and bone scintigraphy (Figure 1). In the only recent report, SPECT of SIJ with calculated indices of uptake had sensitivity of 80% and specificity of 97% for sacroiliitis in 46 patients with chronic low back pain.^9^

### Combined SPECT/CT Imaging

A combination of SPECT and CT has been used in various fields in medicine for functional and anatomical imaging; SPECT/CT has further increased specificity compared with SPECT in clinical practice by conjoining the anatomical information provided by CT and permitting better characterization of equivocal lesions. The SPECT/CT combination has been suggested previously as a useful diagnostic modality for the evaluation of sacroiliac dysfunction.^10^ In a study involving 20 patients with early SpA, diagnosed by Amor criteria, SPECT/CT of SIJ demonstrated reliable reproducibility, sensitivity of 80%, and specificity of 84% for SIJ involvement.^11^ Of importance, low-dose CT, minimizing the radiation exposure, was used in this study.

### Positron Emission Tomography

The [^18^F]FDG positron emission tomography (PET)/CT technique has been examined previously as an additional tool for the diagnosis of enthesitis in SpA patients.^12^ As a modality for the diagnosis of sacroiliitis, [^18^F]FDG PET/CT has been shown to be of little value, with negative results in all 10 patients with ankylosing spondylitis (AS) in one study,^13^ showing inconsistent results in another,^14^ and assessed as not useful for predicting response to TNF-alpha antagonist therapy in a third study.^15^ However, [^18^F]fluoride, which is a bone tracer of osteoblastic activity, was found useful for demonstrating bone activity in AS patients.^13^ Of importance, the lesions detected by [^18^F]fluoride PET/CT did not always correlate with bone marrow edema as seen on MRI, suggesting that this modality may reflect bone formation rather than inflammatory processes in AS patients.^13^,^16^ An additional study examined performance of [^18^F]fluoride PET/CT in 10 patients with non-radiographic axial SpA (nrAxSpA) and 5 patients with AS: PET/CT was reported as positive in all AS patients and negative in all nrAxSpA patients, further suggesting specificity of this imaging for bone formation, rather than for inflammation.^17^ The sensitivity, specificity, and accuracy of [^18^F]fluoride PET/CT for detection of sacroiliitis was calculated as 80%, 77%, and 79%, respectively, in another study, involving 15 AS patients.^18^

### Bone Scintigraphy with Technetium-99m-labeled Anti-TNF-alpha

The first report on the use of monoclonal human anti-TNF-alpha antibody labeled with Tc-99m for the diagnosis of nrAxSpA was published in 2014.^19^ In a study involving 15 patients with axial and peripheral SpA examined with a similar immunoscintigraphic method, radionuclide uptake of anti-TNF-alpha correlated well with clinical, sonographic, and MRI findings.^20^

### Bone Scintigraphy with Technetium-99m-labeled Human Immunoglobulin

Analogous to monoclonal human anti-TNF-alpha antibodies, human immunoglobulin (HIG) is expected to accumulate in the areas of active inflammation and, when labeled with Tc-99m, can be detected by a scanner. The only pilot study using this modality in five AS patients suggested that the method can have value in the diagnosis of acute inflammation of SIJ.^21^

## DISCUSSION

The diagnosis of SpA can be challenging. The combination of a characteristic clinical presentation, elevated C-reactive protein/erythrocyte sedimentation rate, and positive imaging may enable first-visit diagnosis by rheumatologists in many patients. But in others, particularly at the early stage of the disease, the path to the diagnosis is much more complicated. A rheumatologist, in the absence of widely accepted diagnostic criteria for axial SpA, may have to decide whether to adhere strictly to the restrictive 2009 classification criteria^3^ and use them for the diagnosis, or, at least in some patients, to look for additional evidence for disease presence, utilizing tools that are less validated. The latter approach may benefit the patient with an early diagnosis, unachievable by resorting to the too specific and less sensitive classification criteria.

The presence of sacroiliac involvement is a cornerstone in the diagnosis of axial SpA. The positive imaging of sacroiliitis, as a confirmation of suspected SpA, is always looked for by a rheumatologist. As was noted above, at present the spectrum of recommended SIJ imaging modalities to this end is scanty and, in practice, includes only conventional X-rays and MRI. From this viewpoint, the addition of another validated tool for assessing the SIJ would be of primary importance. The present review suggests that bone scintigraphy, particularly SPECT with calculation of indices, or SPECT in combination with low-dose CT can be a sensitive and specific tool for the diagnosis of sacroiliitis, particularly in patients with equivocal or negative MRI examination of SIJ (Table 1). Of course, controlled studies examining the positive and negative predictive values of the suggested algorithm in different subsets of patients with suspected SpA should be performed. However, in our opinion, these radionuclide methods can have a place in the individualized approach to SpA diagnosis and should not be neglected.

**Table 1:** Reported Performance of Radionuclide Methods for the Diagnosis of Sacroiliitis.

Ref.	Method	Patients	Sensitivity (%)	Specificity (%)
6	Quantitative bone scintigraphy with technetium-99m-MDP	207	Any sacroiliitis, 64.9 Bilateral sacroiliitis, 40.2 Unilateral sacroiliitis, 24.7	Any sacroiliitis, 50.5 Bilateral sacroiliitis, 57.7 Unilateral sacroiliitis, 92.8
7	Quantitative bone scintigraphy with technetium-99m-MDP	30	32	100
9	Single-photon emission computed tomography (SPECT)	91	80	95
11	Combined SPECT/CT imaging	20	80	84.6
18	Positron emission tomography (PET)/CT	15	80	77

CT, computed tomography; MDP, methylene diphosphate; SPECT, single-photon emission computed tomography

The contribution of radionuclide methods to future scientific research in this field seems promising as well. Studies using [^18^F]fluoride PET/CT imaging in SpA will, hopefully, help to clarify details of new bone formation in SpA. In addition, immunoscintigraphy, using labeled monoclonal anti-cytokine antibodies, may contribute to the understanding of SpA pathophysiologic processes at different disease stages and in patient subsets, as well as aid individualization of the choice of biological treatment in the near future.

In summary, the spectrum of existing radionuclide tools for the evaluation of patients with SpA—both highly sensitive and specific methods for its diagnosis in the clinical setting and new promising techniques for scientific research—is expanding and promises further progress in the study of axial SpA.

## Abbreviations

AS: ankylosing spondylitis
CT: computed tomography
HIG: human immunoglobulin
MDP: methylene diphosphate
MRI: magnetic resonance imaging
nrAxSpA: non-radiographic axial SpA
PET: positron emission tomography
SpA: spondyloarthritis
SIJ: sacroiliac joint
SPECT: single-photon emission computed tomography
